# A FinFET with one atomic layer channel

**DOI:** 10.1038/s41467-020-15096-0

**Published:** 2020-03-05

**Authors:** Mao-Lin Chen, Xingdan Sun, Hang Liu, Hanwen Wang, Qianbing Zhu, Shasha Wang, Haifeng Du, Baojuan Dong, Jing Zhang, Yun Sun, Song Qiu, Thomas Alava, Song Liu, Dong-Ming Sun, Zheng Han

**Affiliations:** 1 Shenyang National Laboratory for Materials Science, Institute of Metal Research, Chinese Academy of Sciences, Shenyang, 110016 China; 2 School of Material Science and Engineering, University of Science and Technology of China, Anhui, 230026 China; 3 Institute of Chemical Biology and Nanomedicine, State Key Laboratory of Chemo/Biosensing and Chemometrics, College of Chemistry and Chemical Engineering, Hunan University, Changsha, 410082 China; 4 Anhui Key Laboratory of Condensed Matter Physics at Extreme Conditions, High Magnetic Field Laboratory and University of Science and Technology of China, Chinese Academy of Science (CAS), Hefei, Anhui Province China; 5 State Key Laboratory of Quantum Optics and Quantum Optics Devices, Institute of Opto-Electronics, Shanxi University, Taiyuan, 030006 P. R. China; 6 Collaborative Innovation Center of Extreme Optics, Shanxi University, Taiyuan, 030006 P. R. China; 7 Key Laboratory of Nanodevices and Applications, Suzhou Institute of Nanotech and Nanobionics, Chinese Academy of Science, Suzhou, 215123 P. R. China; 8 Université Grenoble Alpes, CEA, LETI, 38000 Grenoble, France

**Keywords:** Two-dimensional materials, Electronic devices

## Abstract

Since its invention in the 1960s, one of the most significant evolutions of metal-oxide-semiconductor field effect transistors (MOS-FETs) would be the three dimensionalized version that makes the semiconducting channel vertically wrapped by conformal gate electrodes, also recognized as FinFET. During the past decades, the width of fin (*W*${}_{{\rm{fin}}}$) in FinFETs has shrunk from about 150 nm to a few nanometers. However, *W*${}_{{\rm{fin}}}$ seems to have been levelling off in recent years, owing to the limitation of lithography precision. Here, we show that by adapting a template-growth method, different types of mono-layered two-dimensional crystals are isolated in a vertical manner. Based on this, FinFETs with one atomic layer fin are obtained, with on/off ratios reaching $\sim\!\! 10^{7}$. Our findings push the FinFET to the sub 1 nm fin-width limit, and may shed light on the next generation nanoelectronics for higher integration and lower power consumption.

## Abstract (web summary)

FinFETs are an evolution of metal-oxide-semiconductor field effect transistors (MOSFETs) featuring a semiconducting channel vertically wrapped by conformal gate electrodes. Here, the authors use a two-dimensional semiconductor to push the FinFET width to sub-nm whilst achieving a 107 ON/OFF ratio.

## Introduction

Field effect transistors, which usually have the architecture of a conduction channel gated through an insulating layer, are known to be the core of modern semiconductor technologies^1^. As depicted by the famed Moore’s law, the number of transistors per unit area in an integrated circuit is expected to be exponentially increasing in a yearly time scale, which requires a continuous reduction of transistor size^2^. Nevertheless, when the channel width becomes at the order of sub-10 nm, performances of the conventional planar-structured FETs are often poisoned by the quantum confinement effects^3^. One way to mitigate this issue is to take advantage of the vertical dimension and fabricate fin-like conduction channel wrapped by oxides and gate electrodes, thus giving rise to a FinFET configuration with smaller device footprint, higher gate efficiency, and lower power consumption^4^.

To date, most of the FinFETs are fabricated by a top-down method, with the conducting fin etched from bulk plane^5^. However, this is forcibly limited by the precision of state-of-the-art lithography tools, while the narrowest line width of which can reach $\sim$6 nm^6^. Yet satisfaction cannot be met with the iterative progresses of semiconducting industry, as the further improvement of optical lithography technique is extremely difficult^7^.

**Fig. 1:**
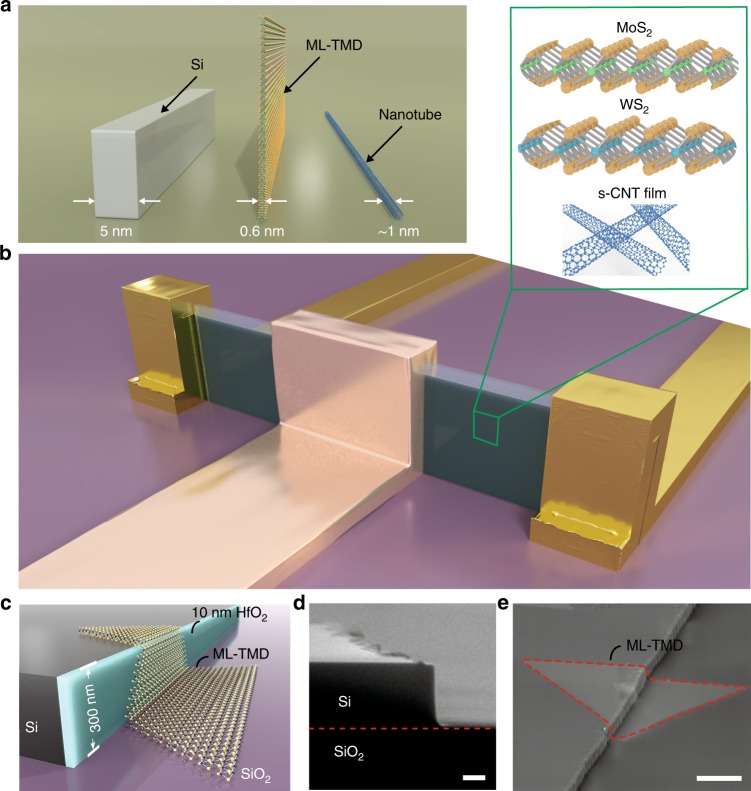
The ML-FinFET with ${W}_{{\rm{fin}}}$ $\sim$0.6 nm. **a** Demonstration of the ML-TMD fin as compared to etched Si-fin and nanotubes in their typical dimensions. **b** Schematic picture of the ML-FinFET presented in this work. Inset in **b** shows the several options for depositing the fin materials in this structure. **c** Schematic of a monolayer MoS${}_{2}$ crystal growing over a 300 nm height Si step, with the side wall coated by HfO${}_{2}$. **d** Scanning electron micrograph (SEM) image of the as-fabricated 300 nm Si step. **e** SEM morphology of a typical monolayered MoS${}_{2}$ crystal growing over the 300 nm height step, while the dash red lines highlight the outline of the crystal. Scale bars in **d**, **e** are 100 nm and 1 µm, respectively.

Recently, efforts have been devoted to novel structure of FETs using low-dimensional materials as a platform. For example, two dimensional (2D) MoS${}_{2}$ planar FET with a single carbon nanotube (CNT) gate was demonstrated, pushing gate lengths of FETs to a sub-1 nm scale^8^. Similarly, CNT can serve as an ultra narrow conducting channel with graphene source and drain^9^. Few-layered semiconducting MoS${}_{2}$^10–14^, as well as CNT films^15–18^, were also utilized to replace the conventional Si channel for developing novel FinFETs. 2D semiconductors are reported to be less pronged to short channel effects, but they however usually take similar space on chips as compared to those conventional Si-based technologies, making them far from satisfactory in terms of scaling^19–21^. The verticallization of monolayer (ML) 2D van der Waals (vdW) materials thus becomes a goal that has been long pursued, as it is not only a prototype that reaches the single atom limit of the Fin width in a FinFET structure, but also can keep the fin height as compared to the etched Si-fins and nanotubes (see Fig. 1a). So far, challenges still remain in terms of experimental realization of vertically free-standing 2D nano flakes.

In this work, we show that by designing a universal template-growth method, different types of ML 2D crystals are isolated in a vertical manner. Based on this bottom-up fabrication route, the vertical free-standing 2D MLs are further conformally coated with insulating dielectric and metallic gate electrodes, forming a 0.6 nm ${W}_{{\rm{fin}}}$ ML-FinFET structure (illustrated in Fig. 1b). A series of ML vdW materials and gate materials are demonstrated to show similar performances, with on/off ratios reaching around 10${}^{7}$. Although, at this stage, these ML-FinFETs still struggle for relatively low-carrier mobilities, the realization of physical limit of ${W}_{{\rm{fin}}}$ in the FinFET structure opens up possibilities for future nanoelectronic applications.

## Results

### Template growth of 2D crystals on a 300-nm height Si step

In order to obtain vertically clamped 2D vdW materials with a considerable height as shown in Fig. 1b, we here devised a template growth method to allow deposition over a few-hundred nm step of materials such as transitional metal dichalcogenide (TMD), as shown in Fig. 1c. First, $\sim$300 nm step edge (Fig. 1d; Supplementary Fig. 1) was etched from a planar Si on insulator wafer (an optimization of the fabrication process to obtain a steep vertical step was performed, see Methods and Supplementary Figs. 2–4). Note that, as indicated in Fig. 1c, there is a 10 nm HfO${}_{2}$ coating on the side wall of the step edge, achieved via atomic layer deposition (ALD) and anisotropic dry etching (we call it a plane-removing process, see Supplementary Fig. 5). Tests were also carried out without the HfO${}_{2}$ coating on the side wall, which failed to yield the desired fin structure (more discussion can be found in Supplementary Fig. 6). This is to help to protect and support the TMD flakes in the consequential fabrication processes. A dedicated wet spray chemical vapor deposition (CVD) process was optimized (see Supplementary Figs. 7–11) to conformally grow ML 2D vdW crystals over the as prepared 300 nm step edges, which serve as vertical templates, as depicted in the schematic picture in Fig. 1e. It is found that this template growth can be a rather universal route to grow TMDs, such as MoS${}_{2}$ and WS${}_{2}$ on those sharp-edge templates, as well as to deposit other thin layer such as CNT-films (see Supplementary Figs. 12 and 13). More experimental details proving the ML-nature of those grown TMD flakes can be found in the Supplementary Figs. 14 and 15.

**Fig. 2:**
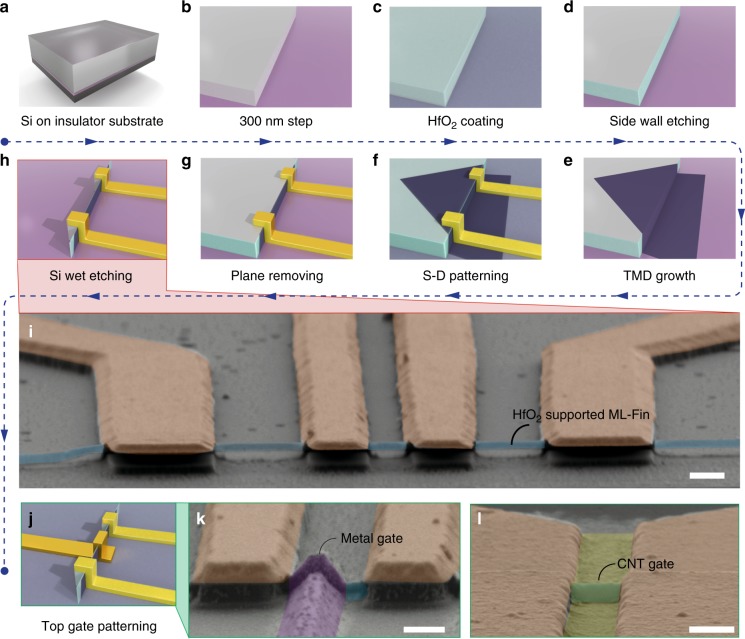
Fabrication of ML-FinFETs. **a**–**h** Schematic images of the detailed process for fabricating the ML-FinFETs, with the name of each step labeled below. **i** Zoomed-in false-colored SEM image of a typical vertically clamped ML MoS${}_{2}$, corresponding to the **h**. **j** Gate electrodes deposited on the HfO${}_{2}$-coated structure in **h**, finishing the whole process of ML-FinFET fabrication. **k**, **l** False-colored SEM images of the ML-FinFET with metal and CNT-film gates, respectively. Scale bars in **i**, **k**, **l** are 500, 500 and 200 nm, respectively.

Before moving to the next steps of nano fabrications of FinFET, we tested the conventional in-plane FET made of the as-grown TMD crystals on SiO${}_{2}$. Characteristic n-type field effect curves are seen in most of the in-plane MoS${}_{2}$ FET samples (more details in Supplementary Fig. 16). According to the aspect ratio in the MoS${}_{2}$ devices, such as illustrated in Fig. 2k, l, room temperature electron mobility was estimated to be at the order of 10 cm${}^{2}$ V${}^{-1}$ s${}^{-1}$. WS${}_{2}$ planar FETs and s-CNT film FETs show similar performances, as shown in Supplementary Figs. 17 and 18. The relatively low mobilities of the planar FETs (Supplementary Figs. 16 and 17) made following this route may be caused by the existence of extra defective scattering centers, owing to the nature of the wet-spray growth. Further improvements are required in the future works. Nevertheless, this growth method provides a unique opportunity for the fabrication of ML-FinFET, as will be discussed in the following text.

### The fabrication processes of ML-FinFETs

To describe in detail the feasibility of FinFET with ML fin, we demonstrate each fabrication step in a work flow, as shown in Fig. 2a–h. The preparation of 300 nm sharp edge with the side wall covered by 10 nm HfO${}_{2}$ are illustrated in Fig. 2a–d. Such 300 nm step edges were fabricated in an array, as shown in Supplementary Fig. 1b, in order to achieve batch fabrication. After the wet-spray of precursor and CVD growth, a certain coverage of ML TMDs can be obtained (Fig. 2e) for source–drain (S–D) electrodes deposition after a HfO${}_{2}$ coating (Fig. 2f). To obtain better contacts of electrodes, we have tested systematically the evaporation angle and thickness, as shown in Supplementary Figs. 19 and 20.

One of the most critical processes is the removal of planar part (defined as the plane-removing process) of the as-grown ML TMDs, which conformally cover the step edge. This is to retain only the 2D materials on the 300 nm side wall with an under layer of previously coated HfO${}_{2}$, as shown in Fig. 2d, g and Supplementary Figs. 5 and 21. In the next step, a wet etch (Fig. 2h) was carried out to remove the 300 nm Si step, making the HfO${}_{2}$/TMD/HfO${}_{2}$ sandwich structure vertically clamped by the S–D electrodes. A false-colored SEM image of the sketch shown in Fig. 2h is given in Fig. 2i, with the vertical blue belt (i.e., the HfO${}_{2}$ supported ML-fin of vertical TMD nanobelt, more details can be seen in Supplementary Figs. 21 and 22) clearly seen. Next, shown in Fig. 2j, a gate metallization procedure was done after an ALD coating of HfO${}_{2}$ gate dielectric on the basis of structure shown in Fig. 2h, i. Here, gate electrodes can be made by either metal deposition (see also Supplementary Fig. 23) or CNT-film deposition (see also Supplementary Fig. 24), as illustrated by the false-colored SEM images in Fig. 2k, l. It is noted that the ML-Fin can in principle consist of ML MoS${}_{2}$, WS${}_{2}$, and other thin films including CNT films, as indicated by the insets in Fig. 1b. Indeed, by replacing MoS${}_{2}$ with WS${}_{2}$ or CNT films, we found that the work flow in Fig. 2a–i a universal bottom-up route for fabricating FinFETs with sub 1 nm ${W}_{{\rm{fin}}}$ as its conduction channel.

## Discussion

**Fig. 3:**
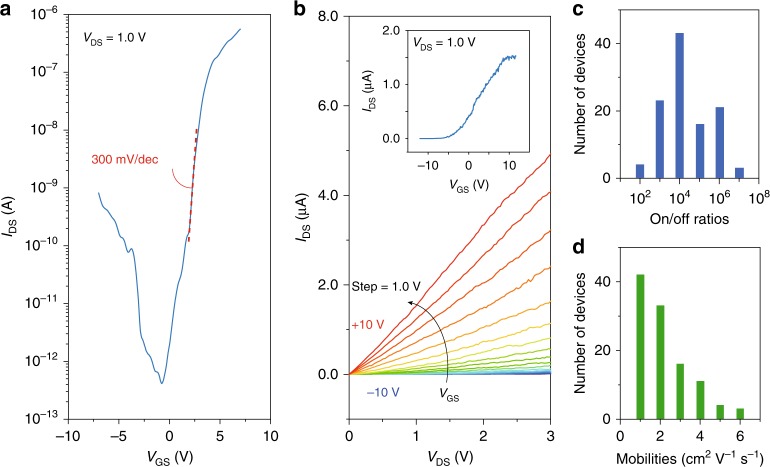
Electrical performances of the MoS${}_{2}$ ML-FinFETs. **a** Field effect curves at 1.0 V source-drain voltage of MoS${}_{2}$ ML-FinFET. **b** $I-V$ curves at different gate voltages for the MoS${}_{2}$ ML-FinFET, the inset figure shows the field effect curve of the same device. **c**, **d** Statistics of on/off ratios and mobilities of MoS${}_{2}$ ML-FinFETs in this work.

In the following text, we discuss the electrical transport of those prepared TMD ML-FinFETs. As shown in Fig. 3a, a typical field effect curve (measured from Sample-108) is shown with the best sub-threshold swing (SS) obtained to be 300 mV per decade. Meanwhile, $I-V$ characteristics of a typical ML-FinFET (measured from Sample-52) are shown in Fig. 3b. Rather linear $I-V$ curves can be seen, with its corresponding field effect curve plotted in the inset. Semiconducting CNT-film FinFET and WS${}_{2}$ ML-FinFETs are also fabricated using the same methods and tested to show similar electrical properties (Supplementary Figs. 24 and 26). Among the tested devices, on/off ratios of those ML-FinFETs are extracted to be in the range of 10${}^{2}$–10${}^{7}$, with the statistical distribution illustrated in Fig. 3c. Meanwhile, mobilities of those tested ML-FinFETs devices are extracted to be in the range of 1–6 cm${}^{2}$ V${}^{-1}$ s${}^{-1}$, as shown in the statistics in Fig. 3d. Optimizing the material growth conditions to improve the intrinsic mobility of the material is our next plan.

**Fig. 4:**
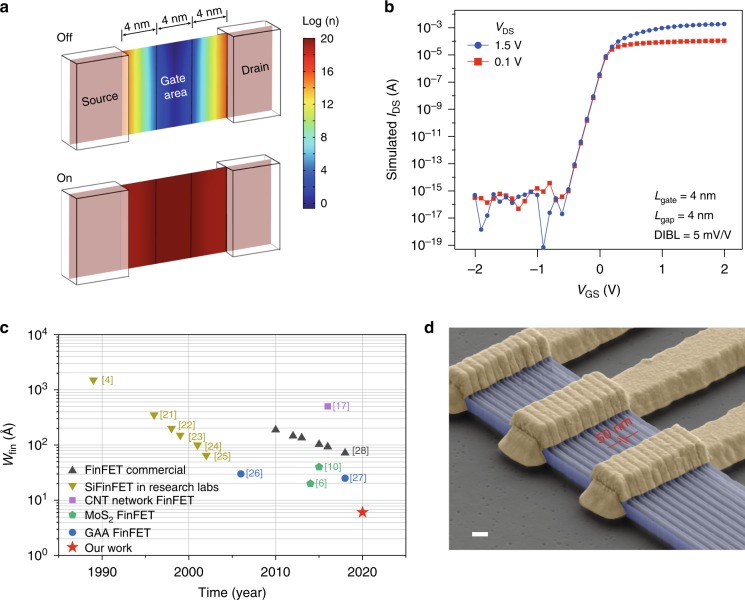
Prospects of the ML-FinFETs. **a** The simulated carrier statics of 4 nm gate length FinFET at off and on states, the color bar represents the carrier density $n$ in a log scale. **b** The simulated field effect curves of 4 nm gate length FinFET at ${V}_{{\rm{DS}}}$ = 0.1 and 1.5 V, respectively. **c** A time scale evolution of ${W}_{{\rm{fin}}}$. Our current work, marked by the red solid star, brings the ${W}_{{\rm{fin}}}$ to the one atomic layer limit, which in principle cannot be shrunk any further. **d** False-colored SEM image of an ML-Fin array, with 50 nm pitch and 300 nm fin height. Scale bar in (**d**) is 300 nm.

At this stage, electrical performances such as SS and mobility of the ML-FinFET can be further improved to meet the criteria for future application. Nevertheless, modelings of a ML-FinFET with 4nm gate length and 0.65 nm ${W}_{{\rm{fin}}}$ were carried out to address the performance of such ideal cases, using the finite element software COMSOL Multiphysics model (Methods). As shown in Fig. 4a, the model device was built with three parts, i.e., channel, source, and drain regions, where the gate area was separated with 2 nm dielectric layer of HfO${}_{2}$. The simulated distribution of carrier density of OFF (${V}_{{\rm{GS}}}$ = −1 V) and ON (${V}_{{\rm{GS}}}$ = 1 V) states at ${V}_{{\rm{DS}}}$ = 0.1 V are shown in Fig. 4a, which correspond to the red dots at ${V}_{{\rm{GS}}}$ = −1 V and 1 V in Fig. 4b. A typical short channel parameter screening length $\lambda$ is computed to be 0.26 nm (Methods). Meanwhile, the on/off ratio and drain-induced-barrier-lowering were estimated to be around $\sim\!\! 10^{11}$ and 5 mV/V, respectively. Figure 4a, b together with these short-channel-effect (SCE) parameters exhibit that, with simulations, a 4 nm gate length FinFET has performances overcoming the SCE with powerful gate controllability. Besides 4 nm gate length ML-FinFET, COMSOL simulations have been done to estimate the performance of variable gate length and dielectric layer thickness (Supplementary Figs. 27–29). Our simulations thus prove that the ML-FinFET can be of much improved performances. As shown in Fig. 4c we plot the iterative progresses of FinFETs as a function of time line. It is seen that, strikingly, the ${W}_{{\rm{fin}}}$ has been levelling off since 2 decades^4,6,10,11,18,22–28^. Our present work brings this nanostructure to a limit of 0.6 nm ML, an order of magnitude thinner than the ${W}_{{\rm{fin}}}$ of state-of-the-art FinFETs. Furthermore, we have also fabricated the TMD fin-array ML-FinFET as shown in Fig. 4d with different fin spacing (Supplementary Fig. 30), the minimum pitch between two fins reaches 50 nm in our experimental conditions (more data in Supplementary Fig. 31). The ML-Fin arrays can in principle be building blocks for future integrated circuits.

In conclusion, we have developed a universal bottom-up method, with which ML-FinFET with a 0.6 nm fin width can be obtained. We tested different types of single layered two-dimensional crystals as the fins of the ML-FinFETs, and observed SS with 300 mV/dec and on/off ratios reaching 10${}^{7}$ in them. Based on our COMSOL simulation results, potentially, ML-FinFET has a great advantage over the Si and Si/Ge GAA devices in overcoming the short channel effect. Computational modelings also suggest that using the ideal conditions, the performance of the TMD ML-FinFET can be further improved. It is known that ${W}_{{\rm{fin}}}$ has been levelling off at a few nanometers since almost 2 decades, owing to the limitation of lithography precision. Our present work pushes the ${W}_{fin}$ of FinFET to the one atomic physical limit, which is one order of magnitude smaller than the state-of-art devices. It is believed to shed light on the development of next generation nanoelectronics for lower power consumption and higher integration.

## Methods

### Wet spray CVD technique

Preparation of precursor: 20.6 mg Na${}_{2}$MoO${}_{4}$ (99$\%$, Aladdin) or 88.2 mg Na${}_{2}$WO${}_{4}$ (98$\%$, Adamas) dissolved into 10 mL DI water to make precursor solution, respectively. Substrate rinsed by O${}_{2}$ plasma (CIF Tech Co., Ltd. CPC-C-40 kHz) to improve the hydrophilicity. The substrate first coated 0.01 M Na${}_{2}$MoO${}_{4}$/0.03 M Na${}_{2}$WO${}_{4}$ solution was then placed into the center of the furnace. Crucible with 80 mg S powder (99.5$\%$, Sigma Aldrich) 10 cm upstream the heating center. Before heating, the tube was flushed with 300 sccm Ar for 10 min to keep an inert environment. Then the furnace was heated to 850 ${}^{\circ }$C in 45 min and was kept at this temperature for 40 min with 280 sccm Ar to realize a molten liquid precursor state. Finally, the reaction was finished within 2 min by introducing the sulfur powder into the heating zone for the growth of MoS${}_{2}$. For the growth of WS${}_{2}$, 270 sccm Ar and 30 sccm H${}_{2}$ mixture gases were introduced to convey the sulfur vapor and other growth conditions were same as that of MoS${}_{2}$. After growth, the furnace was naturally cooled down to room temperature.

### Semiconducting CNT and metallic CNT deposition

Semiconducting CNTs: the high-purity ($$> $$99.9$$\%$$) semiconducting CNTs was mixed with toluene by a volume ratio of 1:15 and then ultrasonically treated for 10 min. Before the deposition process, a SOI substrate was preheated at 130 $${}^{\circ }$$C for 20 min. Then, a ML of hexamethyldissilazane (MCC Primer) was spin-coated onto the substrate to improve the surface wettability. Subsequently, the substrate was immersed in the semiconducting CNT solution at 60 $${}^{\circ }$$C for 3 h and then settled statically for 6 h at room temperature. Finally, the substrate was successively soaked in the toluene, acetone and isopropyl alcohol for 5 min, and heated at 150 $${}^{\circ }$$C for 30 min. Metallic CNTs: the TUBALL_TM_ SWCNTs used as gate electrodes were purchased from OCSiAl Inc. The dispersant 9-(1-octylonoyl)-9H-carbazole-2,7-diyl (PCz) were prepared by Suzuki polycondensation. The deposition process is the same with the semiconducting one.

### Nano fabrications

SOI wafer with 300 nm device thickness is used as the substrate in our experiment. The SOI wafer is first patterned into different size arrays using photolithography (ABM/6/350/NUV/DCCD/M) process and ICP-RIE (Plasma Pro 100 Estrelas). After that, 10 nm HfO${}_{2}$ is deposited at 150 ${}^{\circ }$C using ALD (Savannah S100). RIE (RIE-10NR) is used to etch the planar part materials. The electrodes are fabricated with standard EBL process and evaporated with electron beam evaporation (ei-501z).

### Electrical measurement setups

The fabricated devices were characterized using an optical microscope (Nikon LV100ND), SEM (FEI XL30 SFEG using an accelerating voltage of 10 kV) and AFM (Bruker Dimension Icon AFM). The electrical performance of the FinFETs was measured using a semiconductor analyzer (Agilent B1500A) and a probe station (Cascade Microtech Inc. 150-PK-PROMOTION) under ambient conditions.

### Finite element simulation

The finite element simulation basing on the commercial software COMSOL Multiphysics^29^ was performed, where semiconductor module basing on Maxwells equation, Boltzmann transport theory together with Neumann boundary conditions were emplied^30–32^. Three-dimensional model of FinFET (Fig. 4a) was built with gate around the gate area by three sides, including the top and two side walls. The gap between gate and source/drain region was set to be 4 nm. Here, the source and drain regions were heavily doped (${n}$ = $1{0}^{20}$ cm${}^{-3}$) to make them conducting, and the channel region (gated and gapped regions) was slightly N-doped with (${n}$ = $1{0}^{8}$ cm${}^{-3}$ (6.5 cm${}^{-2}$)) for model simulation converging. COMSOL simulation was performed at 300 K, and the material properties used in COMSOL simulation were shown in Supplementary Table 1. ${W}_{{\rm{Fin}}}$ was set to be 0.65 nm as the thickness of ML MoS${}_{2}$, and the gate length were simulated at 200, 100, 50, and 4 nm, respectively. The Shockley–Read–Hall was used to simulate the trapping assisted recombination^33^. Dielectric layer HfO${}_{2}$ thickness is set to be 2 and 20 nm. The source and drain contacting are set to be ohmic. Screening length was calculated^34^ using

1 $$\lambda =\sqrt{({\varepsilon }_{{\rm{Si}}{{\rm{O}}}_{2}}/{\varepsilon }_{{\rm{Hf}}{{\rm{O}}}_{2}}){W}_{{\rm{Si}}{{\rm{O}}}_{2}}{W}_{{\rm{Hf}}{{\rm{O}}}_{2}}},$$

where the $\varepsilon$ represents the relative dielectric constant, and $W$ is the thickness.

## Supplementary information

Supplementary Information

## Data Availability

The data that support the findings of this study are available at Zenodo (2020), 10.5281/zenodo.3672715.
