# Supplementary material for: A FinFET with one atomic layer channel
Source: Nat Commun. 2020 Mar 5;11:1205. doi: 10.1038/s41467-020-15096-0 (PMC7058032; doi:10.1038/s41467-020-15096-0)
Supplement: Supplementary file 1 — Supplementary Information [file 41467_2020_15096_MOESM1_ESM.pdf]

Supplementary Materials for

A FinFET with one atomic layer channel

Chen, Sun, and Liu, *et al.*

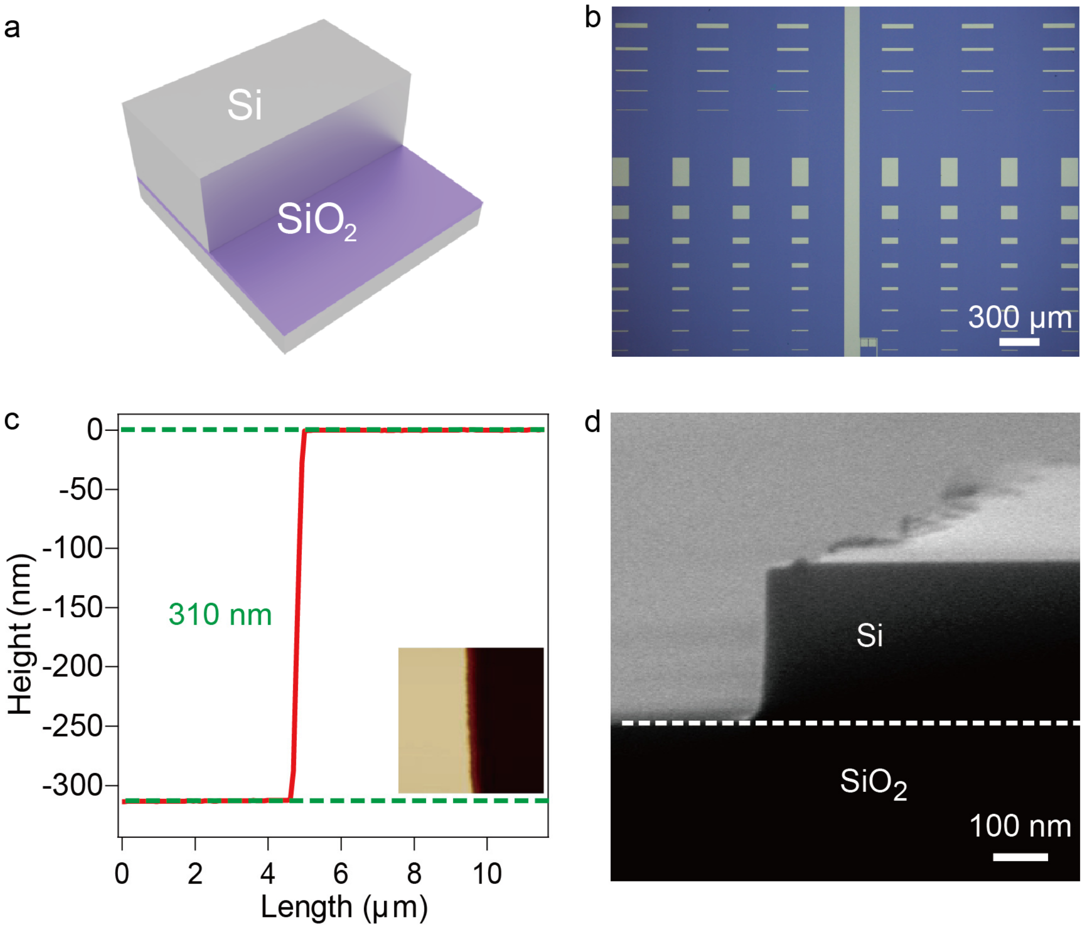

Supplementary Figure 1. The structure of Si-step array. (a) Schematic diagram of the Si step. (b) Optical image for different sizes of Si step arrays. (c) AFM image of the height for the Si step. (d) SEM image for the cross section of the Si step.

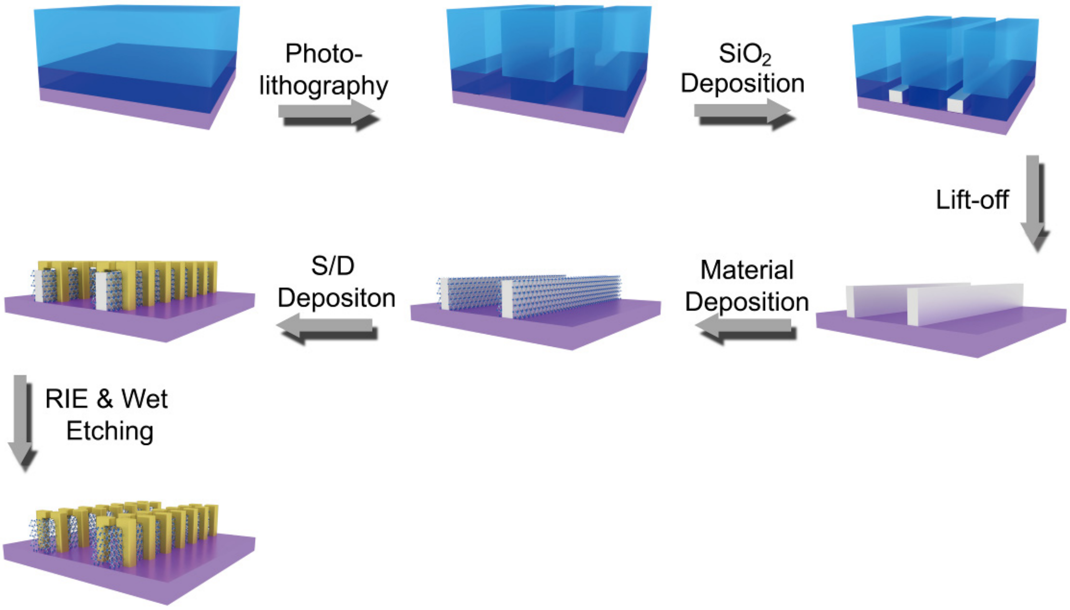

Supplementary Figure 2. The fabrication flow diagram of WS_2 FinFET on SiO_2 step substrate using the initial method. 200 nm SiO_2 is deposited by standard lithograph process followed by using magnetron sputtering method. Then single layer WS_2 is synthesized by using CVD method shown in fig. S11. Then gold electrodes are evaporated using standard EBL process. Finally, SiO_2 is wet etched within NaOH solution. (The material is exposed outside during the Reactive-ion etching (RIE) and wet etching process, which may be the reason for the sample damage.) The details of this method will be shown in the following Supplementary Figure 3, 4 and 6.

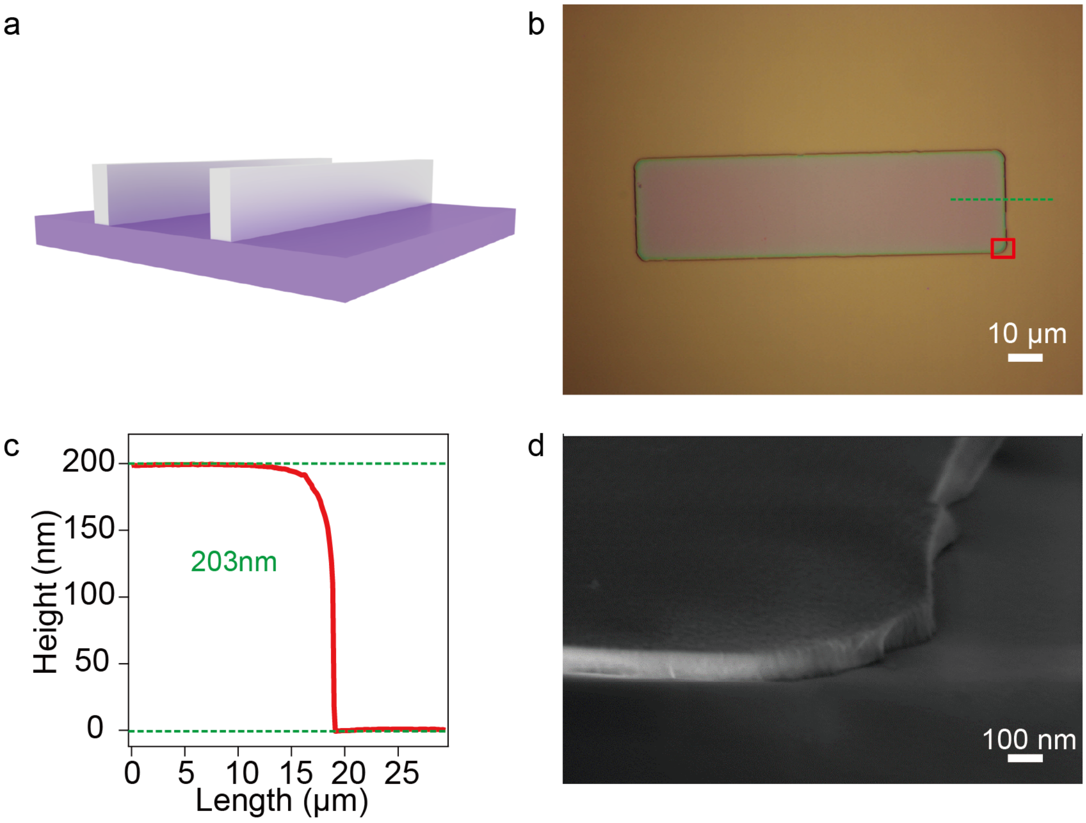

Supplementary Figure 3. The characterization of SiO_2 steps. (a) Schematic diagram of SiO_2 steps. (b) Optical image for a SiO_2 step. (c) AFM line scanning (height profiles) image along the green line from left to right shown in (b). (d) Side view of SEM image (80-degree inclination) for the red rectangle section shown in (b).

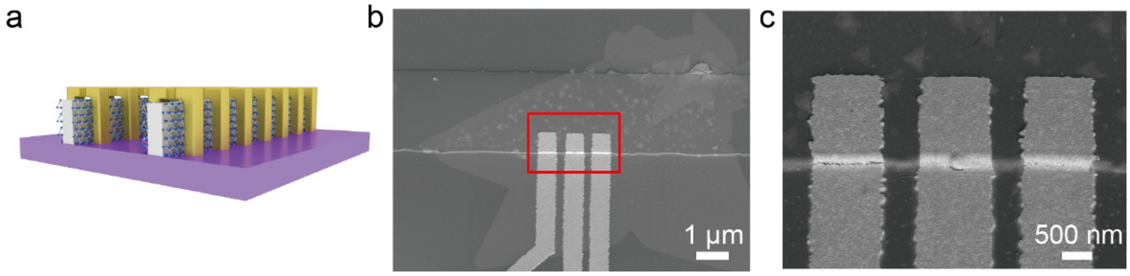

Supplementary Figure 4. Source/Drain (S/D) electrodes on the SiO_2-steps. (a) Schematic diagram of the S/D electrodes on the SiO_2 steps. (b) SEM image (30-degree inclination) of the S/D electrodes on the SiO_2 step. (c) Magnification SEM image for the red rectangle section shown in (b), which shows that WS_2 is continuously and uniformly grown across the step.

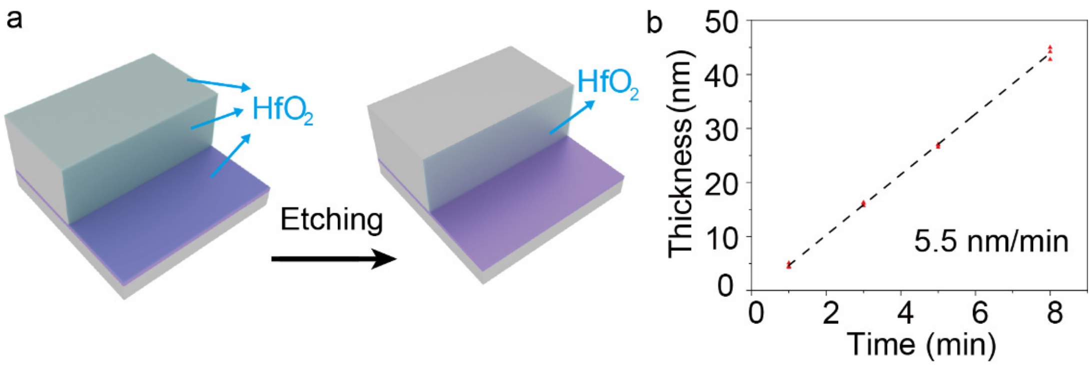

Supplementary Figure 5. The etching process of the plane-removing of HfO_2. (a) Schematic diagram of the planar HfO_2 etching process using RIE, only side-wall of HfO_2 is left after etching. (b) The thickness of HfO_2 for different etching time with etching rate of 5.5 nm/min.

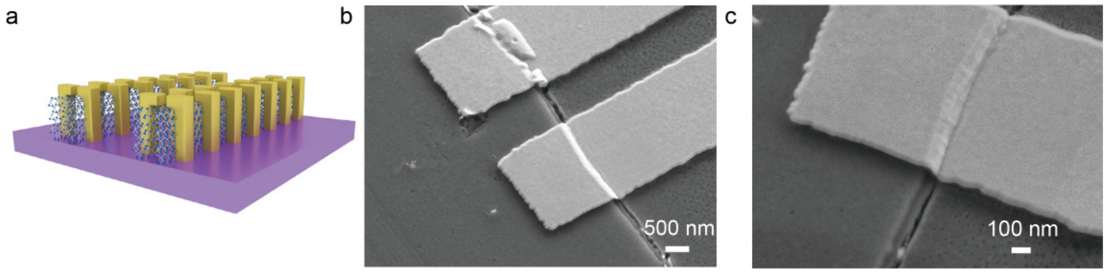

Supplementary Figure 6. Wet-etching of the remaining SiO_2-step. (a) Schematic diagram of the structure after RIE and SiO_2 steps are wet etched. (b) SEM Optical image (30-degree inclination) for the S/D electrodes and WS_2 nano-fin structure. (c) Magnification SEM image for the same sample shown in (b) but with a different perspective. It shows that most part of the WS_2 nano-fin was etched away, and its device was still not conductive, since the SiO_2 sidewalls did not stand vertically enough.

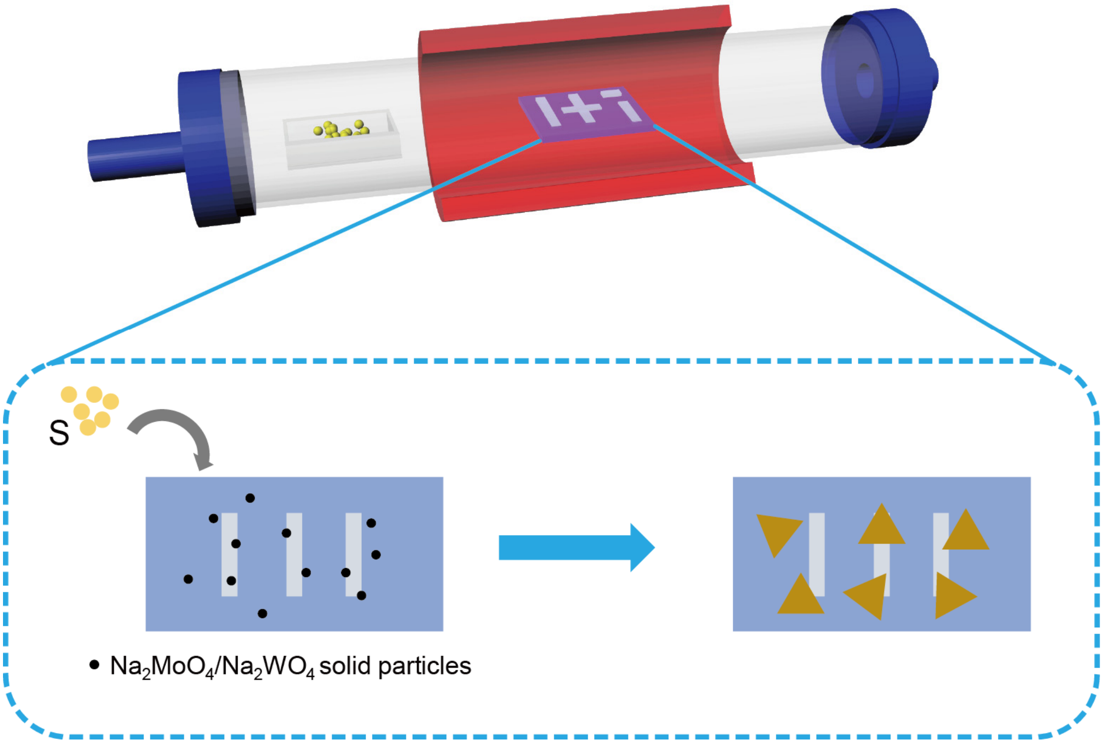

Supplementary Figure 7. The CVD setup for MoS_2 and WS_2 synthesis. Schematic of CVD process and zoomed-in growth process diagrams before and after depositing, which are shown in the dotted blue rectangle. The circles in gold and grey represent the S and Na_2MoO_4/Na_2WO_4 precursor, respectively. The orange triangles indicate the deposited samples.

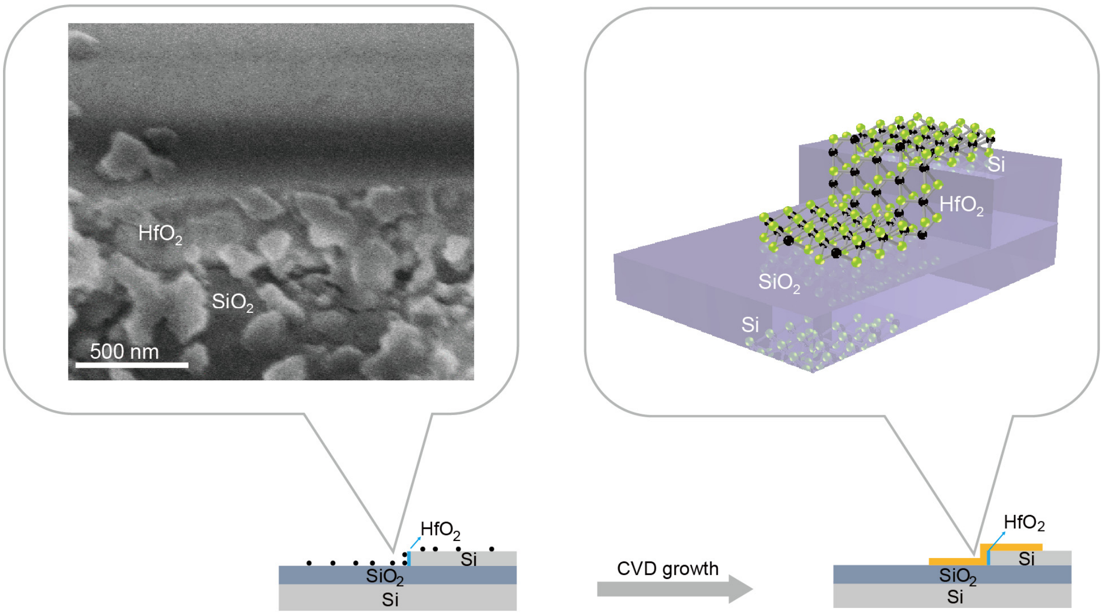

Supplementary Figure 8. Schematics of the growth mechanism of monolayered TMDs. The substrates before (left) and after (after) TMDs growth. The enlarged figures marked by the black squares show the SEM image near the step in (left) and schematic of the step after sample deposition, respectively.

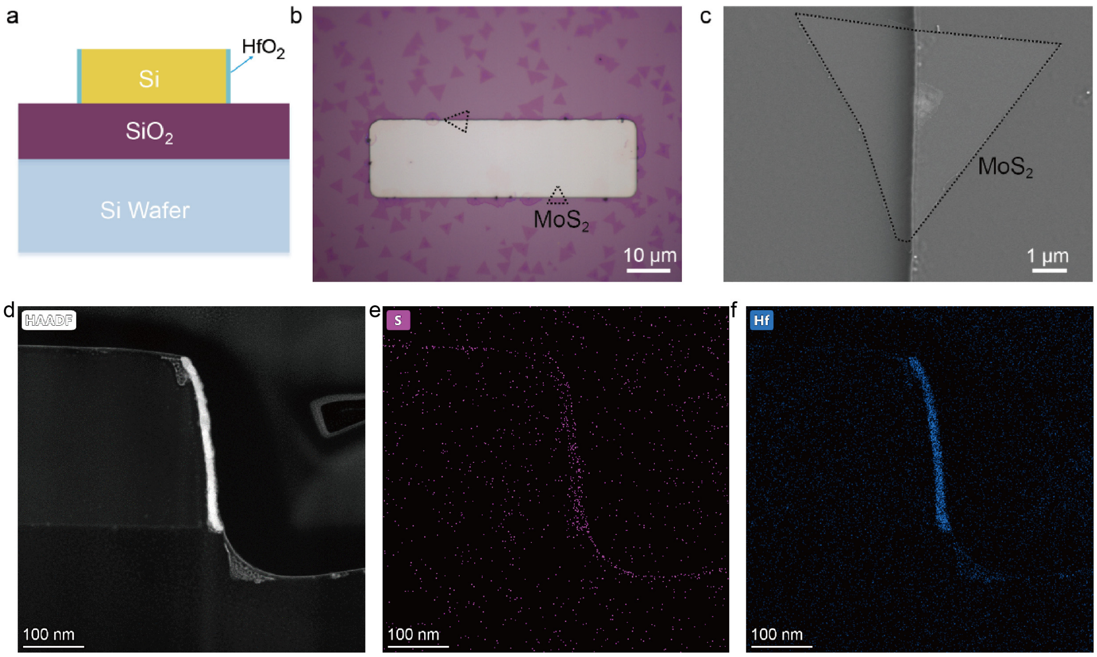

Supplementary Figure 9. CVD synthesized MoS_2 on HfO_2 coated sidewall. (a) Schematic diagram of CVD synthesis substrate. (b) Optical image of few layer single-crystal MoS_2 marked by the black dotted lines grown on both SiO_2 (purple) and Si (yellow) substrate plane. (c) SEM image of few layer single-crystal MoS_2 marked by the dotted black lines continuously grown across the HfO_2 coated sidewalls. (d) TEM cross-section shows the continuous growth of monolayer TMD over the HfO_2 side wall. (e) and (f) are EDS mapping of S and Hf element, respectively. The TEM specimen was fabricated using a sample at the stage of (e) in Figure 2 in the main text.

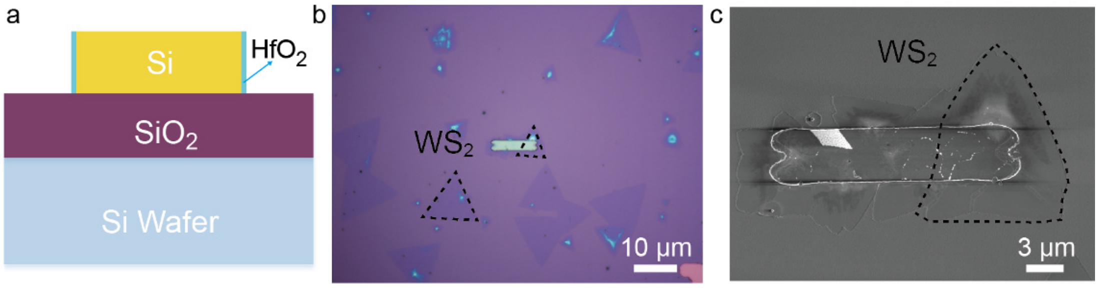

Supplementary Figure 10. CVD synthesized WS_2 on HfO_2 coated sidewall. (a) Schematic diagram of CVD synthesis substrate with HfO_2 coated sidewall. (b) Optical image of few layer single-crystal WS_2 marked by the black dotted lines grown on both SiO_2 (purple) and Si (yellow) substrate planes. (c) SEM image of few layer single-crystal WS_2 continuously grown across the HfO_2 coated sidewall.

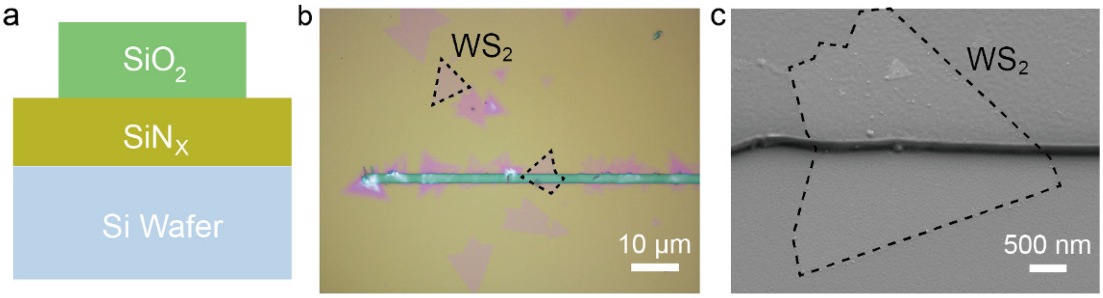

Supplementary Figure 11. CVD synthesized WS_2 on SiO_2 sidewall. (a) Schematic diagram of CVD synthesis substrate. (b) Optical image of few layer WS_2 (pink) marked by the black dotted lines grown on both SiN_x (yellow) and SiO_2 (green). (c) Scanning electron microscope (SEM) image of a few layer single-crystal WS_2, which is marked by the black dotted lines continuously grown across the SiO_2 sidewall.

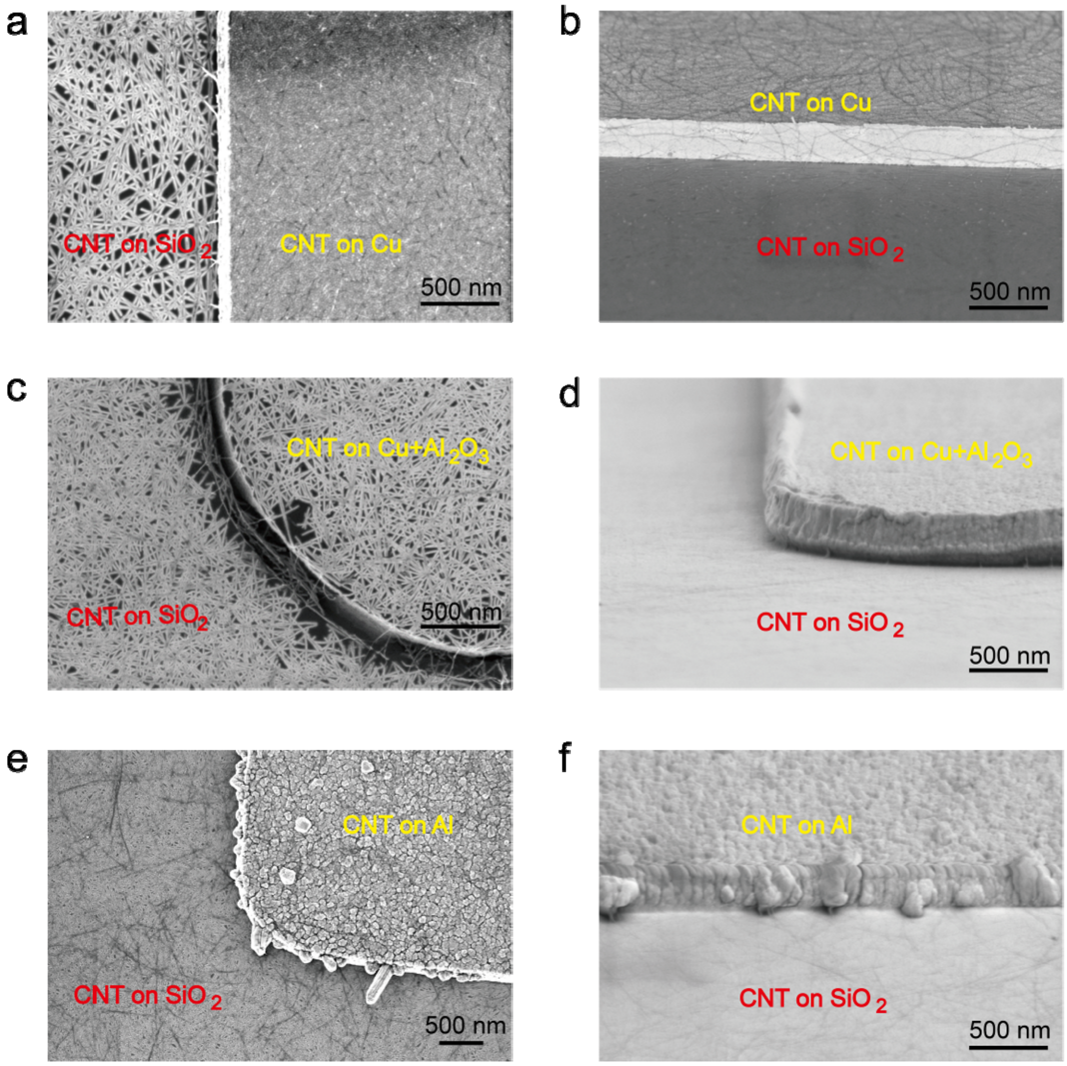

Supplementary Figure 12. SEM images for the CNT deposited on metal substrates. Top (a) and side (b) views of SEM images for the CNT deposited on 400 nm height Cu step. The SEM images for the top (c) and side (d) views of the CNT deposited on the 400 nm Cu covered by 50 nm Al_2O_3 step. SEM images for the top (e) and side (f) views of the CNT deposited on the 400 nm Al step.

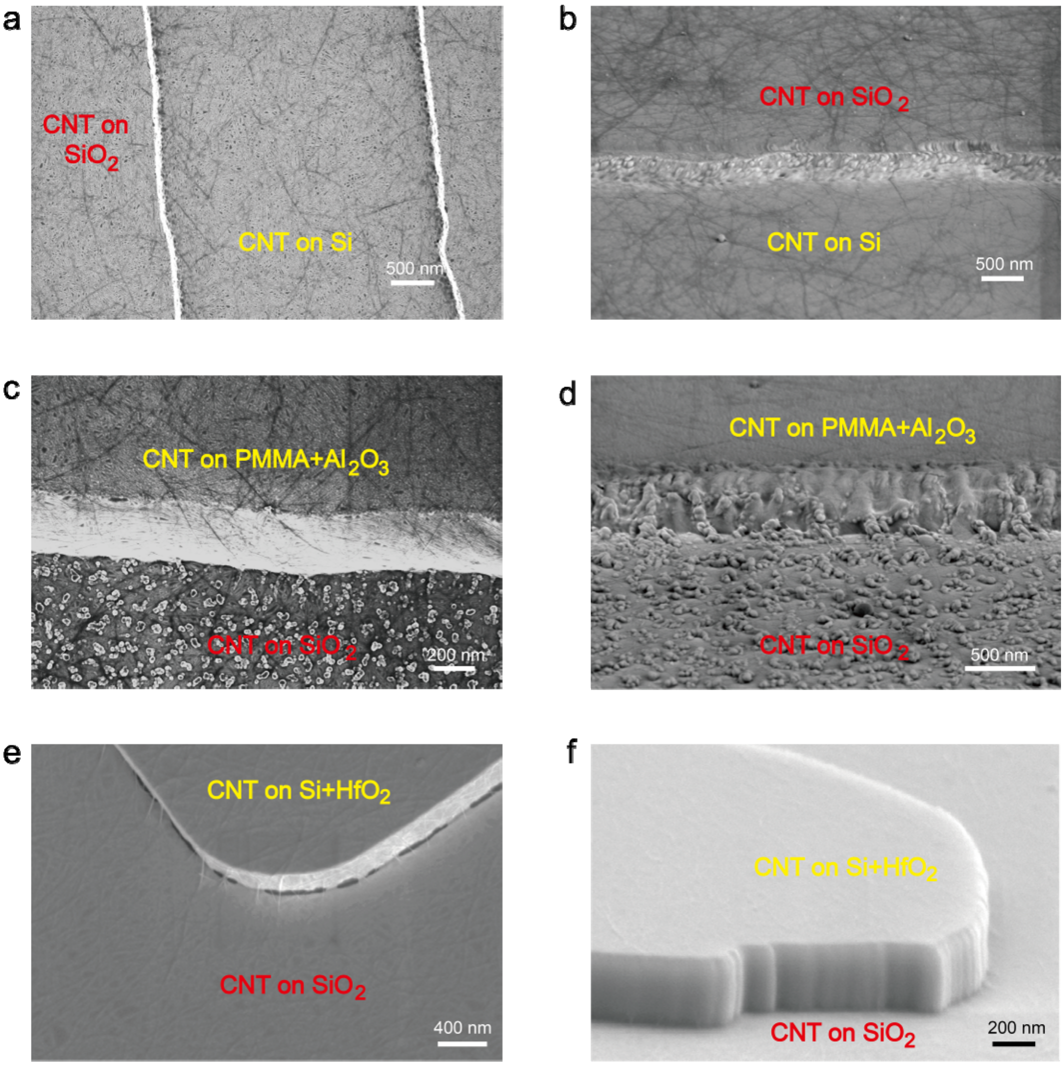

Supplementary Figure 13. SEM images for the CNT deposited on non-metal substrates. The SEM images for the top (a) and side (b) views of the CNT deposited on the 200 nm SiO_2 step. The SEM images for the top (c) and side (d) views of CNT deposited on the 600 nm PMMA covered by 30 nm Al_2O_3 step. The SEM images for the top (e) and side (f) views of CNT deposited on the 300 nm Si covered by 10 nm HfO_2 step.

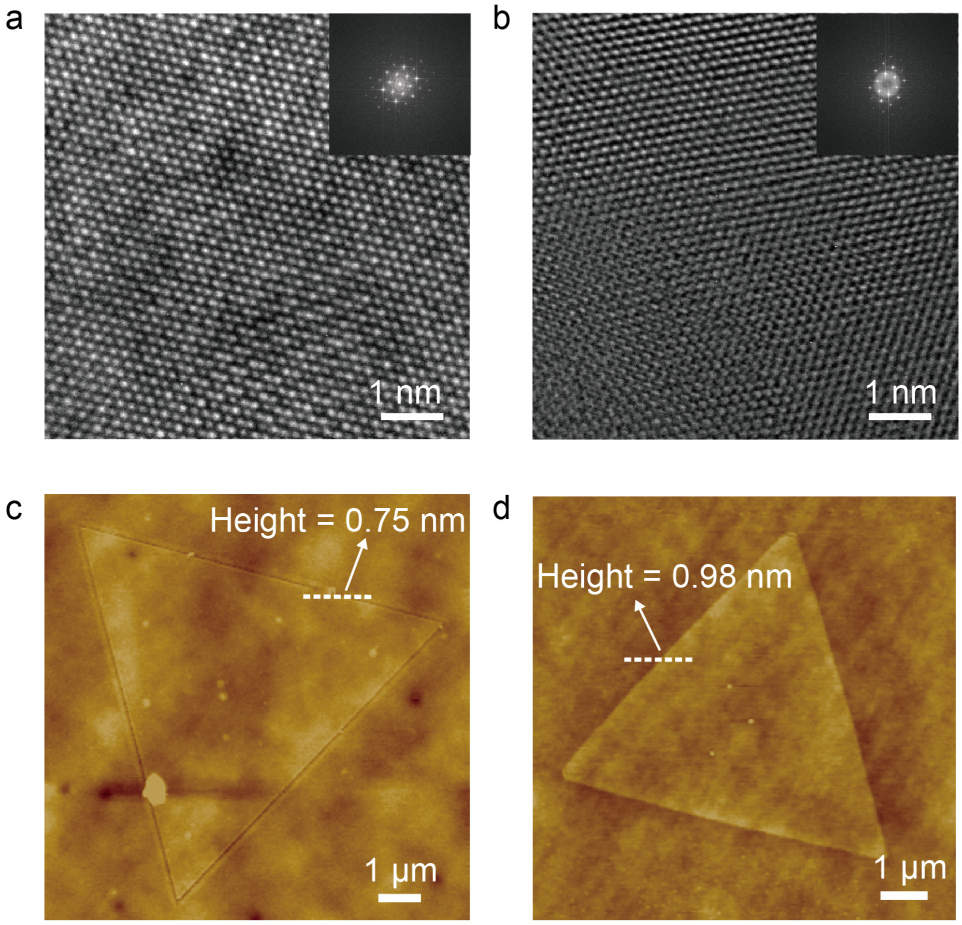

Supplementary Figure 14. High-resolution transmission electron microscopy (HRTEM) and atomic force microscopy (AFM) images of the TMDs. HRTEM and inset SAED images of single layer MoS_2 (a) and WS_2 (b), respectively. AFM mappings of single layer MoS_2 (c) and WS_2 (d), respectively.

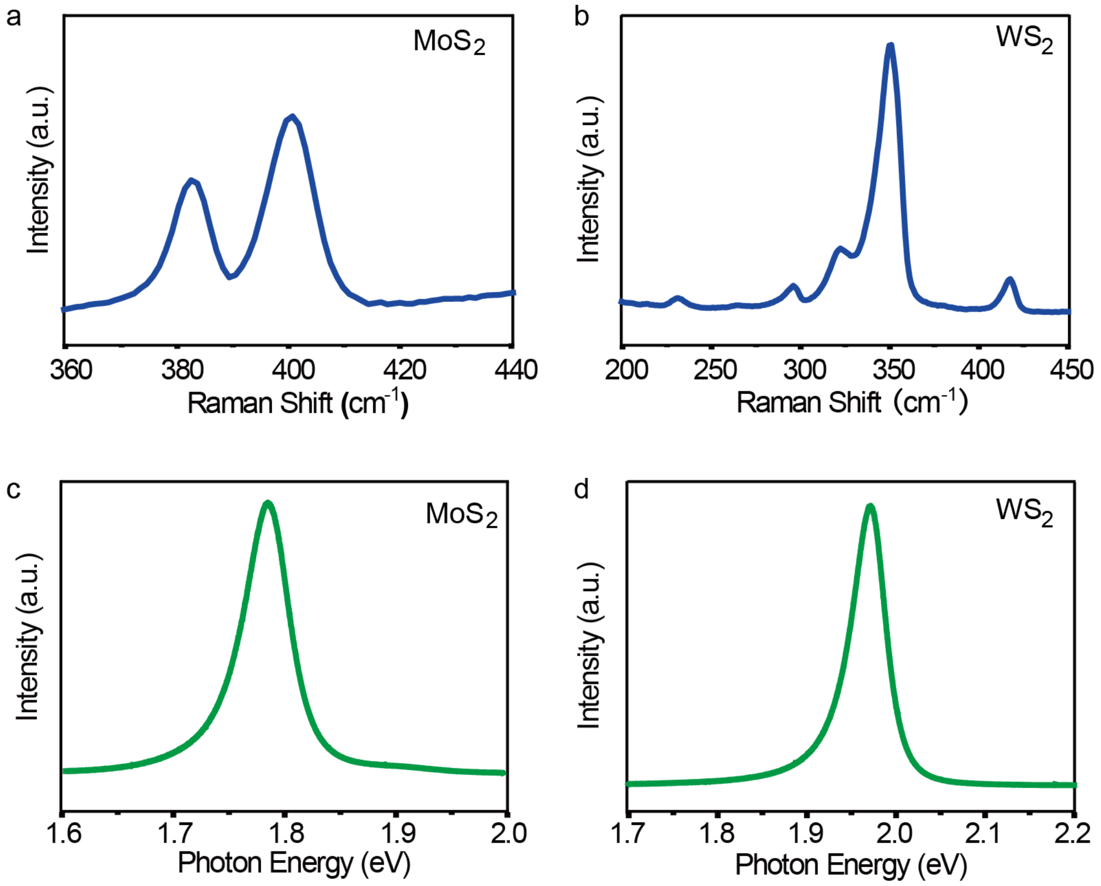

Supplementary Figure 15. Raman spectroscopy and Photoluminescence (PL) spectrum of the TMDs. Raman spectrums of single layer MoS_2 crystal (a) and WS_2 (b), respectively. PL spectrums of single layer MoS_2 (c) and WS_2 (d), respectively.

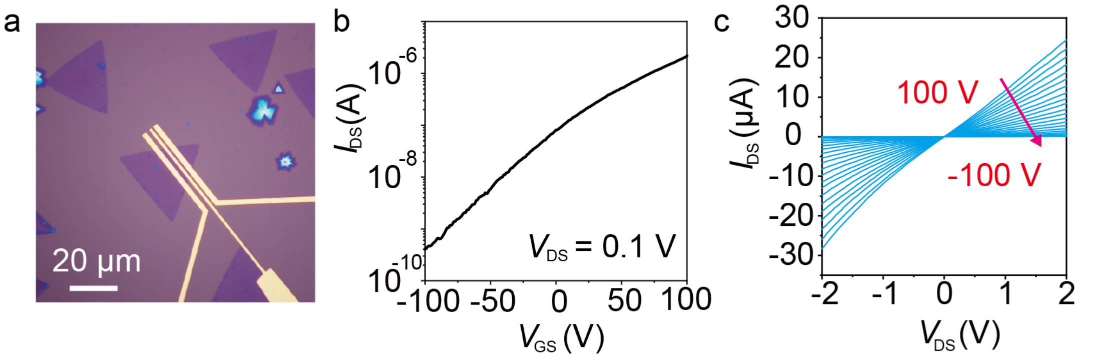

Supplementary Figure 16. Planar MoS_2 FET. (a) Optical image of the conventional in-plane MoS_2 FET made of the as-grown MoS_2 on SiO_2. The triangle represents our single layer TMD sample, and the gold bars show the deposited electrodes. (b) Transfer characteristic curve for the device shown in (a) at room temperature, the source-drain voltage is set to be 0.1 V. (c) Output characteristic curves measured at the same device in (a), while the gate voltage V_{GS} was swept from -100 to 100 V shown by the red arrow in the figure.

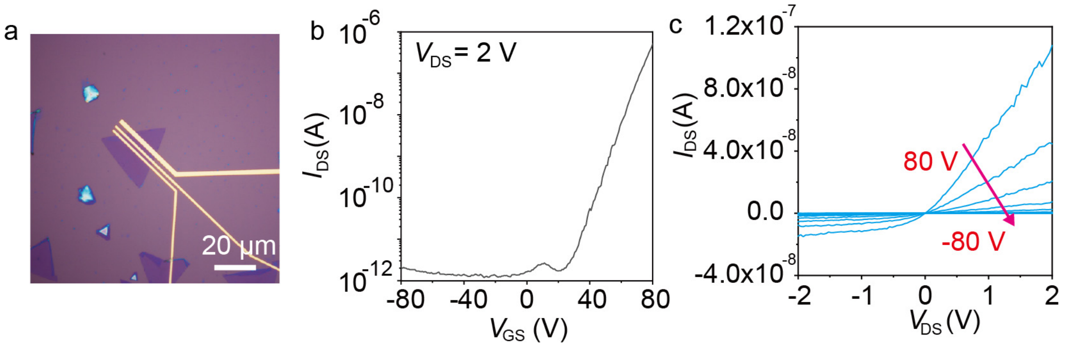

Supplementary Figure 17. Planar WS_2 FET. (a) Optical image of the conventional in-plane WS_2 FET made of the as-grown WS_2 on SiO_2. The triangle represents our single layer TMD sample, and the gold bars show the deposited electrodes. (b) Transfer characteristic curve for the device shown in (a) at room temperature, the source-drain voltage is set to be 2 V. (c) Output characteristic curves measured at the same device in (a), while the gate voltage V_{GS} was swept from -80 to 80 V shown by the red arrow in the figure.

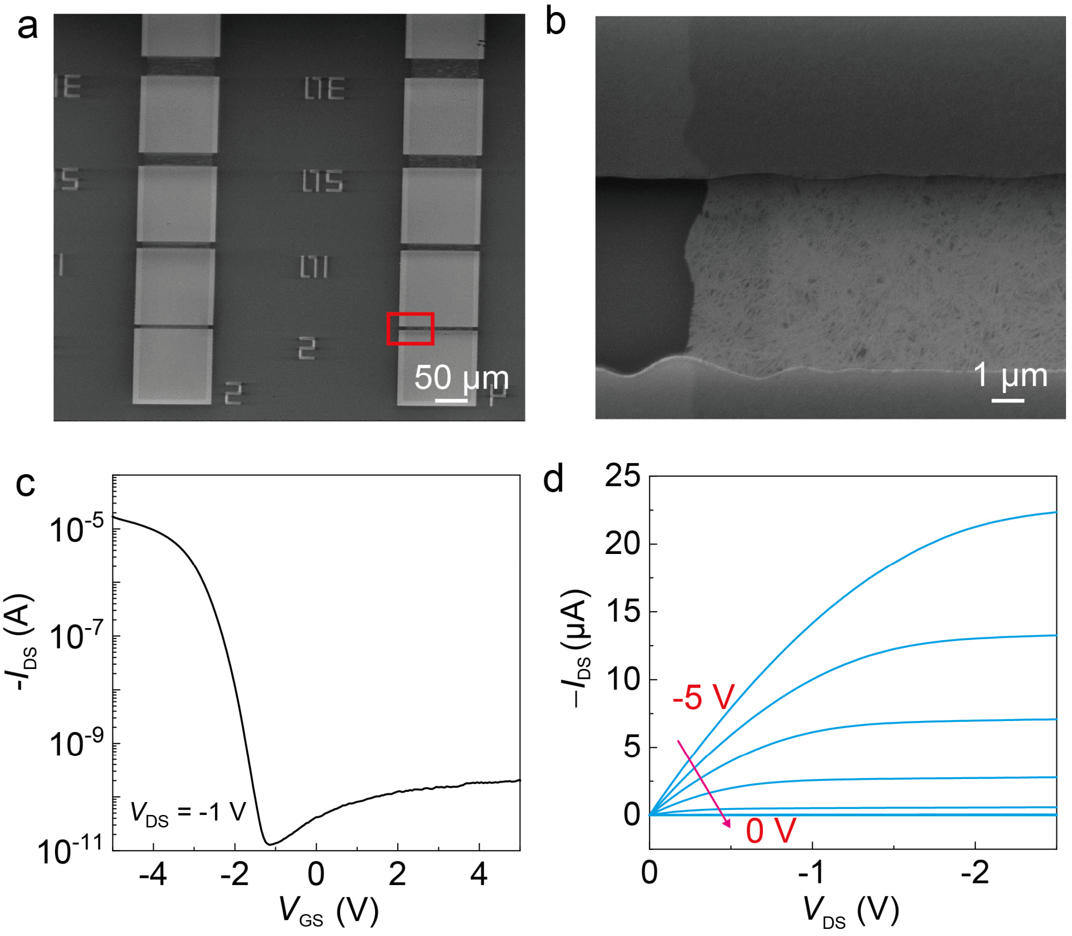

Supplementary Figure 18. Planar CNT-film FET. (a) SEM image of the conventional CNT-film FET made of the semiconducting CNT solution on SiO_2 with different channel sizes. (b) Magnification SEM image for the red rectangle section shown in (a), which shows that CNT-film with high density. (c) Transfer characteristic curve for the device shown in (b) at room temperature, the source-drain voltage is set to be -1 V. (d) Output characteristic curves measured for the same device in (b), while the gate voltage V_{GS} was swept from -5 to 0 V shown by the red arrow in the figure.

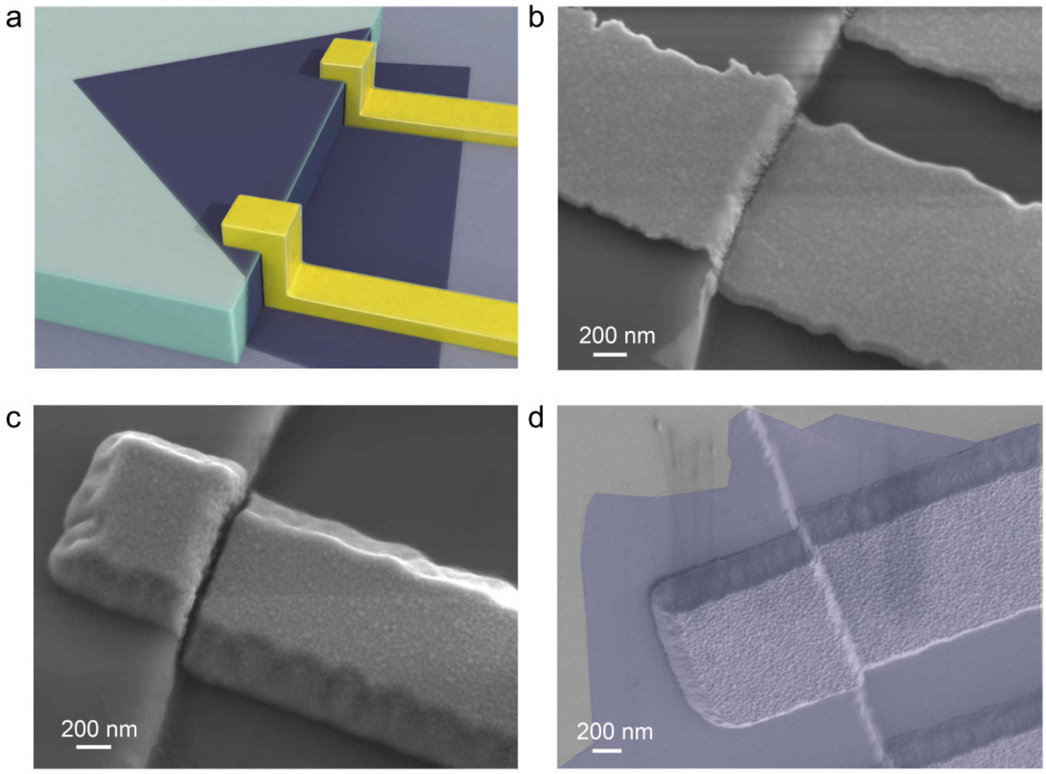

Supplementary Figure 19. S/D electrodes with different thicknesses and evaporation directions. (a) Schematic diagram of the S/D electrodes connected to the sidewall. (b) SEM image for the vertical evaporation 55 nm thickness electrodes. (c) SEM image for vertical evaporation electrodes with 255 nm thickness. (d) SEM image for the tilted evaporation electrodes with 255 nm thickness, the purple area shows the single layered MoS_2.

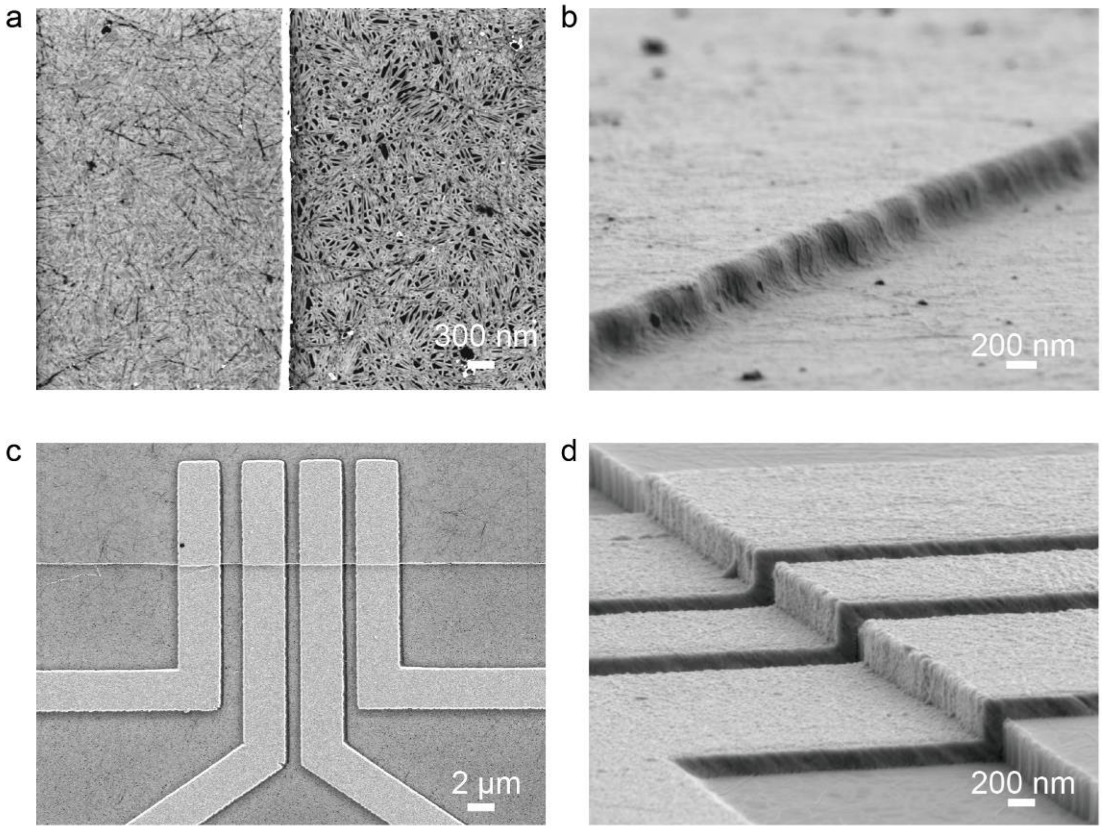

Supplementary Figure 20. CNT-films and the S/D electrodes. (a) and (b) are the SEM images at top and 80-degree inclination views for the morphology of the semiconducting CNT film covering the Si sidewalls, respectively. (c) and (d) are the SEM images at top and 80-degree inclination views for the morphology of the S/D electrodes connected by the CNT films.

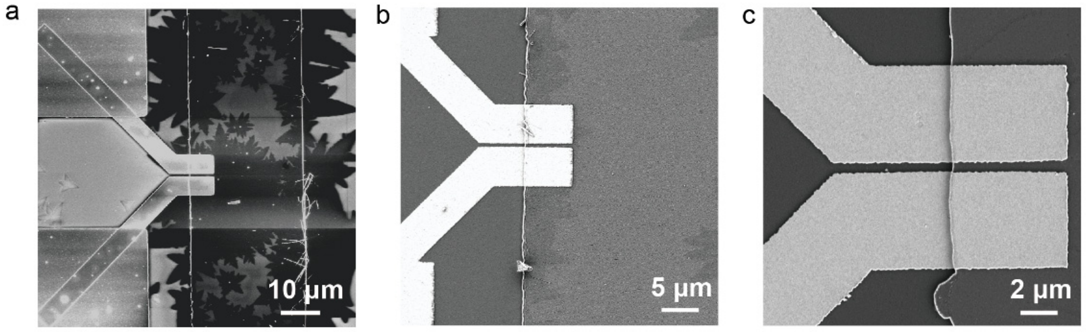

Supplementary Figure 21. MoS_2 ML-FinFET fabrication process. (a) SEM image for the 255 nm thickness S/D electrodes connecting the MoS_2 across the sidewall. (b) SEM image for the MoS_2, while the planar part was etched away by RIE. (c) SEM image for device, while the Si step was wet etched by TMAH solution.

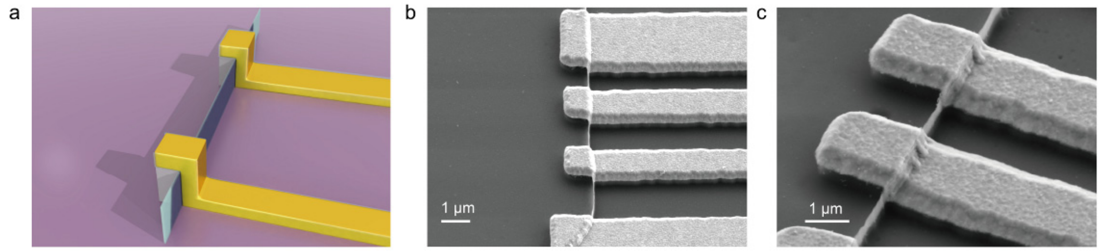

Supplementary Figure 22. ML-fin structures after wet etching. (a) Schematic diagram of the ML-fin after wet etching. (b) and (c) are the SEM images for different views of the ML-fin vertically standing after wet etching.

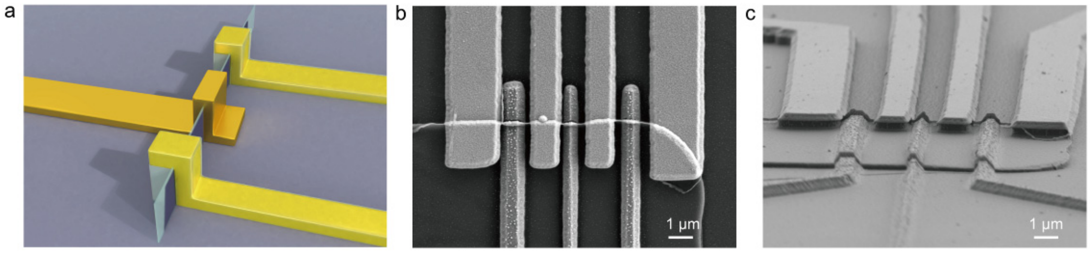

Supplementary Figure 23. Characterizations of the ML-FinFETs with Metal Gates. (a) Schematic diagram of the ML-FinFET with metal gates. (b) and (c) are SEM images at different angles of the gate electrodes overlapping the channel of the device.

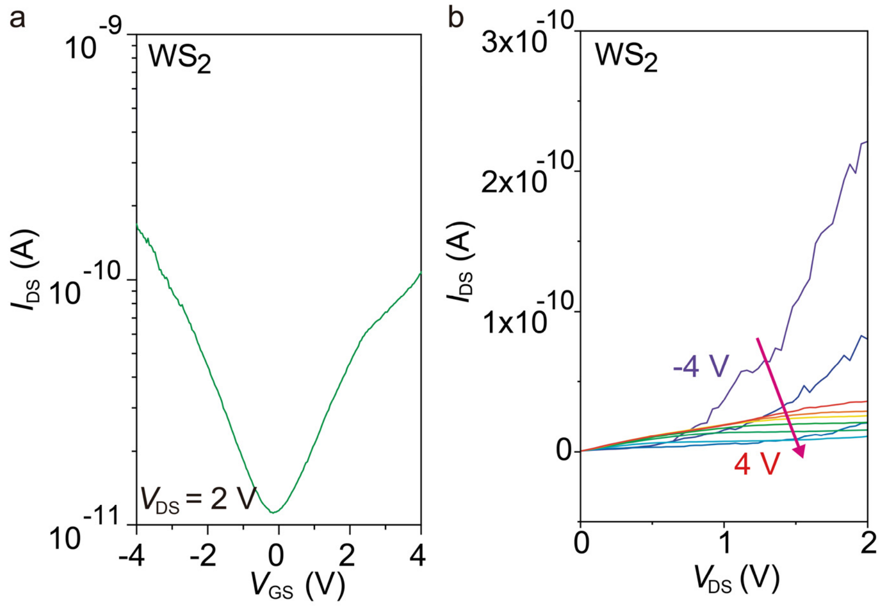

Supplementary Figure 24. Electrical performances of WS_2 ML-FinFET. (a) Transfer characteristic curve under V_{DS} = 2 V for the WS_2 ML-FinFET shown in Fig. S23. (b) *I-V* curves for the same WS_2 FinFET in (a) with V_{GS} sweeping from -4 to 4 V.

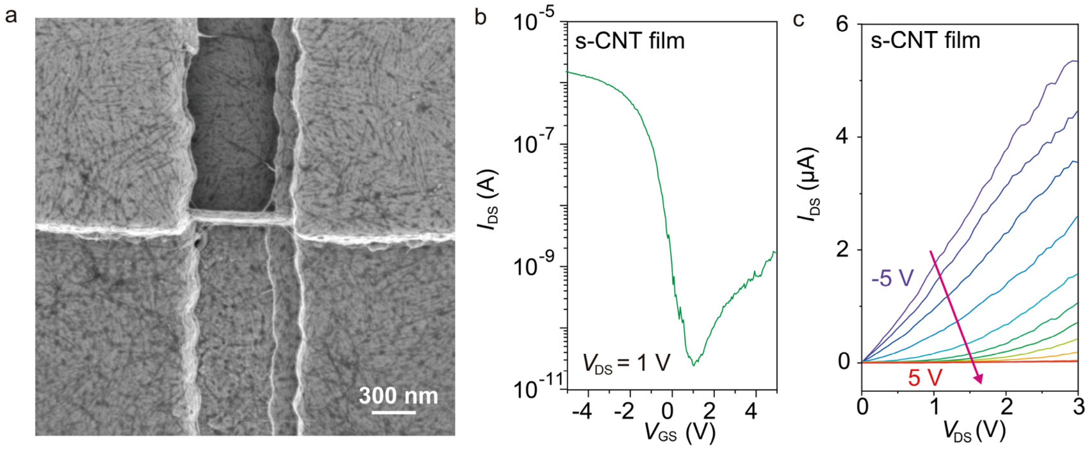

Supplementary Figure 25. CNT-film FinFET with CNT-film gate and its electrical performances. (a) SEM image for the morphology of the semiconducting CNT film fin covered by metallic CNT films as the gate. (b) Transfer characteristic curve of the CNT-film FinFET in (a). (c) Output characteristic curves of the CNT-film FinFETs in (a) with the V_{GS} sweeping from -5 to 5 V.

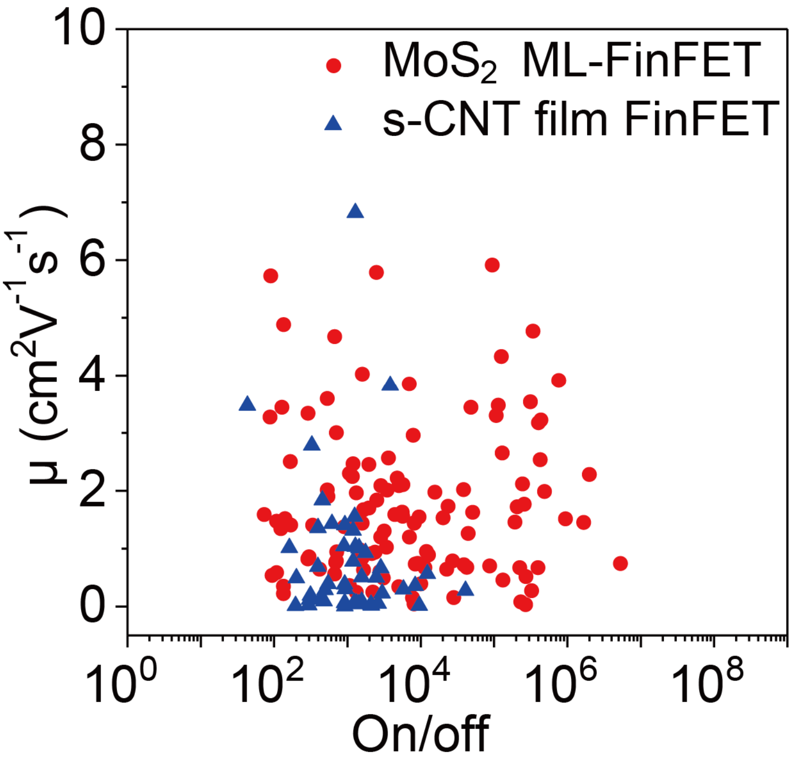

Supplementary Figure 26. Statistics of mobilities μ and on/off ratios of ML-FinFETs. More than one hundred of our MoS_2 ML-FinFET and semiconducting CNT-film-FinFETs devices are tested and the maximum on/off ratio and mobility are 10^7 and 6 $cm^2V^{-1}s^{-1}$, respectively.

Supplementary Table 1. The material properties used in COMSOL simulations.

W_{fin} (nm)[1]	E_{gap} (eV)[2]	$\varepsilon_{\mathrm{MoS_2}}$ (relative)[3]	$\varepsilon_{\mathrm{HfO_2}}$ (relative)[4]	m_e^* (m_0)[5]	m_h^* (m_0)[5]	χ (eV)[6]	μ_e ($cm^2V^{-1}s^{-1}$)[7]	μ_h ($cm^2V^{-1}s^{-1}$)[7]
0.65	1.89	3.93	25	0.37	2.8	4	130	270

Here, W_{fin} is the thickness of fin channel,
E_{gap} is the band gap of monolayer MoS_2,

$\varepsilon_{\mathrm{MoS_2\,(HfO_2)}}$ is the relative dielectric constant of single layer MoS_2(HfO_2),

m_e^* (m_h^*) is the electron (hole) effective mass of MoS_2 single layer,
χ is the electron affinities of monolayer MoS_2,

μ_e (μ_h) is the electron (hole) mobilities.

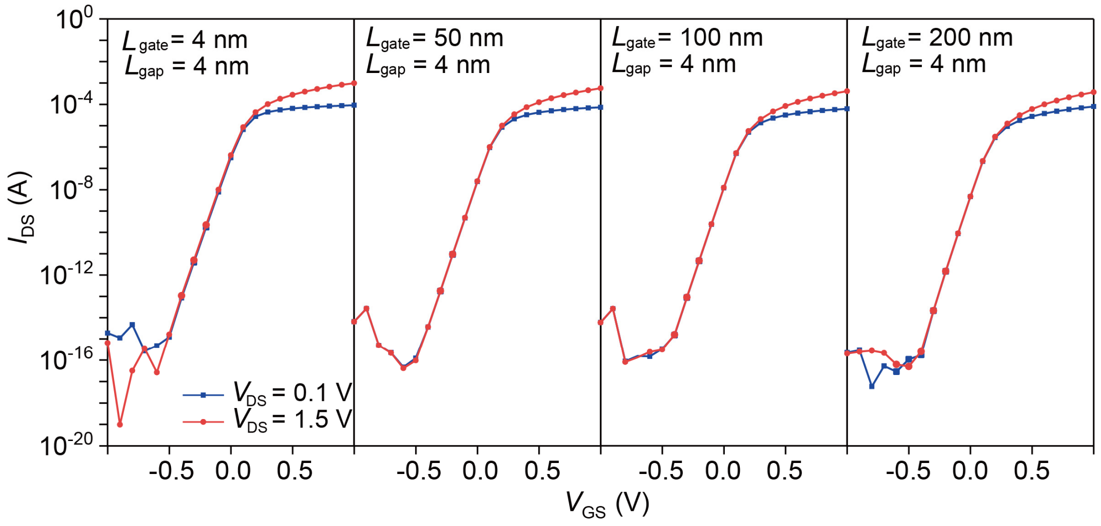

Supplementary Figure 27. COMSOL simulated Fin-FETs with different gate lengths. The field effect curves from COMSOL results for different gate lengths (L_{gate}) at V_{DS} = 0.1 and 1.5 V, respectively.

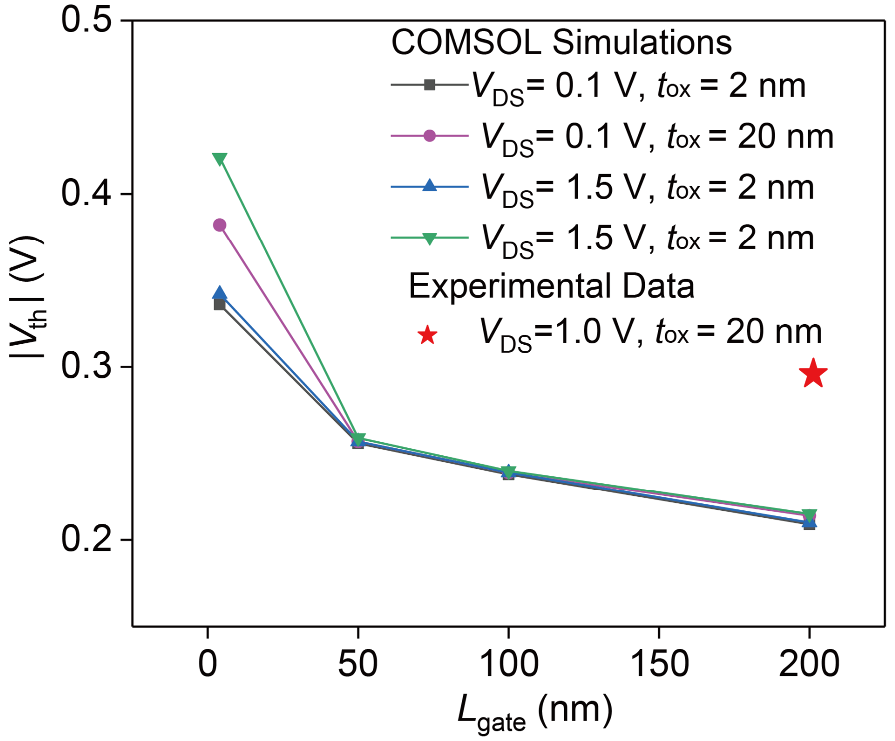

Supplementary Figure 28. $|V_{th}|$ calculated from the simulations. The $|V_{th}|$ are obtained by extracting the V_{GS} at $I_{DS}= 10^{-12}$ A. The experimental data was obtained in the device shown in Figure 3a in the main text.

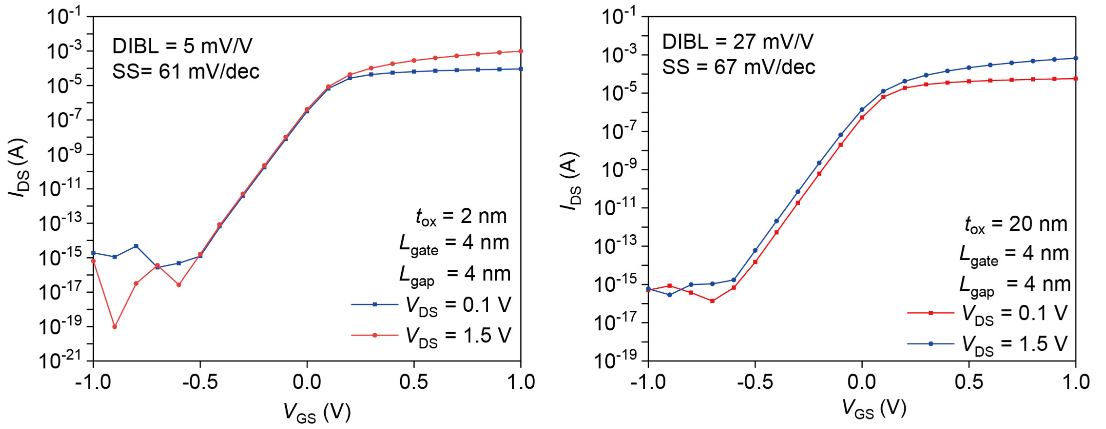

Supplementary Figure 29. COMSOL simulated Fin-FETs with different thickness of the dielectric material. The field effect curves of L_{gate} = 4 nm ML-FinFET for 2 and 20 nm dielectric thickness, respectively.

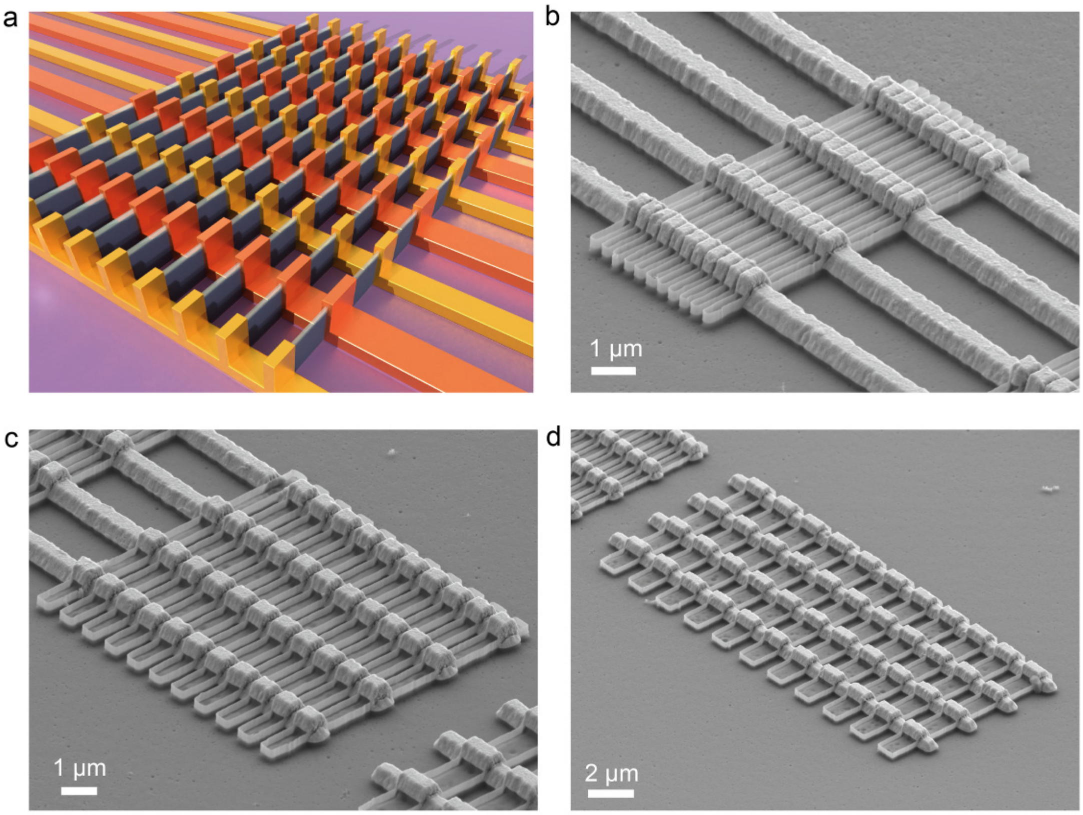

Supplementary Figure 30. Field effect transistors made of ML-Fin arrays. (a) Schematic of a FET with ML-Fin-array. (b)-(d) SEM images of MoS_2 Fin arrays with different fin spacings and channel lengths.

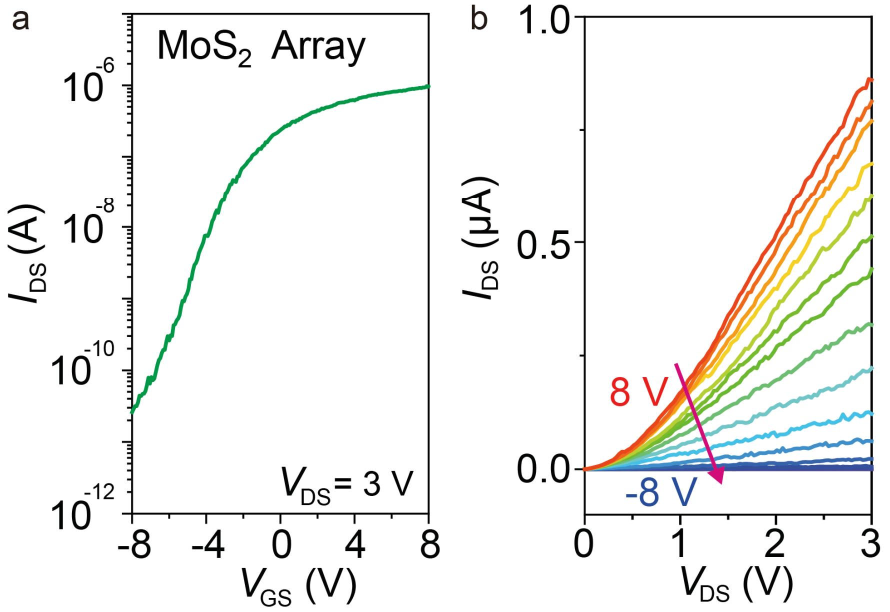

Supplementary Figure 31. Electrical transport performances of a typical FET with the MoS_2 ML-fin-array. (a) Transfer characteristic curve under V_{DS} = 3 V for the MoS_2 FET with ML-Fin-array shown in Supplementary Figure 29. (b) *I-V* curves for the same MoS_2 FET with ML-Fin-array in (a) with V_{GS} sweeping from 8 to -8 V.

References

[1] Y. Li, A. Chernikov, X. Zhang, A. Rigosi, H. M Hill, A. M van der Zande, D. A. Chenet, E. M. Shih, J. Hone, T. F. Heinzs, Measurement of the optical dielectric function of monolayer transition-metal dichalcogenides: MoS_2, $MoSe_2$, WS_2, and WSe_2. Phys. Rev. B 90, 205422 (2014).

[2] K. F. Mak, C. Lee, J. Hone, J. Shan, T. F. Heinz, Atomically thin MoS□: A new direct-gap semiconductor. Phys. Rev. Lett. 105, 136805 (2010).

[3] X. Chen, Z. Wu, S. Xu, L. Wang, R. Huang, Y. Han, W. Ye, W. Xiong, T. Han, G. Long, Y. Wang, Y. He, Y. Cai, P. Sheng, N. Wang, Probing the electron states and metal-insulator transition mechanisms in molybdenum disulphide vertical heterostructures. Nat. Commun. 6, 6088 (2015).

[4] J. Robertson, High dielectric constant oxides. Eur. Phys. J. Appl. Phys. 28, 265–291 (2004).

[5] H. Peelaers and C. G. Van de Walle, Effects of strain on band structure and effective masses in MoS_2. Phys. Rev. B 86, 241401(R) (2012).

[6] S. L. Howell, D. Jariwala, C. C. Wu, K. S. Chen, V. K. Sangwan, J. Kang, T. J. Marks, M. C. Hersam, L. J. Lauhon, Investigation of Band-Offsets at Monolayer–Multilayer MoS_2 Junctions by Scanning Photocurrent Microscopy. Nano Lett. 2015, 15, 4, 2278-2284 (2015).

[7] X. Li, J. T. Mullen, Z. Jin, K. M. Borysenko, M. Buongiorno Nardelli, and K. W. Kim, Phys.Rev. B 87, 115418 (2013).
